# Resolution of Two Steps in Botulinum Neurotoxin Serotype A1 Light Chain Localization to the Intracellular Plasma Membrane

**DOI:** 10.3390/ijms222011115

**Published:** 2021-10-15

**Authors:** Alexander Gardner, William H. Tepp, Marite Bradshaw, Joseph T. Barbieri, Sabine Pellett

**Affiliations:** 1 Department of Microbiology and Immunology, Medical College of Wisconsin, 8701 Watertown Plank Rd, Milwaukee, WI 53226, USA; agardner@mcw.edu; 2 Department of Bacteriology, University of Wisconsin-Madison, 1550 Linden Dr, Madison, WI 53706, USA; whtepp@wisc.edu (W.H.T.); marite.bradshaw@wisc.edu (M.B.)

**Keywords:** botulinum neurotoxin, botulinum neurotoxin serotype A, subtype, toxins, cellular microbiology, SNAP-25

## Abstract

Botulinum neurotoxin serotype A (BoNT/A) is the most potent protein toxin to humans. BoNT/A light chain (LC/A) cleavage of the membrane-bound SNAP-25 has been well-characterized, but how LC/A traffics to the plasma membrane to target SNAP-25 is unknown. Of the eight BoNT/A subtypes (A1–A8), LC/A3 has a unique short duration of action and low potency that correlate to the intracellular steady state of LC/A, where LC/A1 is associated with the plasma membrane and LC/A3 is present in the cytosol. Steady-state and live imaging of LC/A3-A1 chimeras identified a two-step process where the LC/A N terminus bound intracellular vesicles, which facilitated an internal α-helical-rich domain to mediate LC/A plasma membrane association. The propensity of LC/A variants for membrane association correlated with enhanced BoNT/A potency. Understanding the basis for light chain intracellular localization provides insight to mechanisms underlying BoNT/A potency, which can be extended to applications as a human therapy.

## 1. Introduction

Botulinum neurotoxins (BoNTs) are the most potent toxin to humans, and are produced by several species of *Clostridia* [1,2]. BoNTs are ~150 kDa, zinc-dependent metalloproteases, single-chain proteins that are cleaved into disulfide-linked di-chain proteins, by host or native proteases [3,4,5,6]. N-terminal light chain (LC) contains the zinc-binding motif (H-E-X-X-H) and encodes the metalloprotease. There are seven immunologically distinct BoNT serotypes, A–G, which cleave unique soluble N-ethylmaleimide-sensitive factor attachment protein receptor (SNARE) at unique sites [7]. C-terminal heavy chain (HC) comprises a LC translocation domain (HCN) and a host cell receptor-binding domain (HCC) [8,9,10,11].

BoNT HCC/A binds neurons and is internalized by activity-dependent pathways where BoNT/A HCC binds to a polysialoganglioside, such as GT1b, enriched on the presynaptic extracellular membrane surface, followed by association with synaptic vesicle protein 2 (SV2) [12,13,14]. During activity-dependent entry via the SV, the BoNT/A-receptor complex is sequestered within the lumen of the forming SV [15]. Neurotransmitter loading into the developing SV lumen is coupled to lumen acidification, which protonates BoNT/A and triggers the insertion of the translocation domain, HCN, into the SV membrane [16]. HCN/A insertion into the membrane creates a pore of ~15 Å in diameter to facilitate LC translocation into the cytosol of the neuron [17,18]. Once in the reducing environment of the cytosol, the disulfide bond between LC-HCN is reduced by the thioredoxin reductase–thioredoxin system [19], and LC/A refolding is aided by the HCN along with chaperone proteins, such as HSP90 [16,20]. BoNT/A-LC (LC/A) traffics via an unknown mechanism to localize at the plasma membrane [21] to cleave the plasma-membrane-associated synaptosomal-associated protein of 25 kDa (SNAP-25) [22,23]. A subsequent study also reported the activity -independent entry of BoNT/A that used fibroblast growth factor receptor 3 [24].

BoNT/A comprises eight published subtypes, A1–A8, which are neutralized by serotype-A-specific antisera, cleave SNAP-25 at the same site, and possess >84% sequence identity [25,26,27]. BoNT/A subtypes cause the most severe and long-lasting botulism in humans [28]. Among the eight BoNT/A subtypes, BoNT/A3 is unique in possessing a short duration of action and low potency [29], where LC/A3 (A3) is uniquely present in the cytosol, not localized on the plasma membrane as observed for LC/A1 (A1) [23]. Structural analyses revealed a region of particularly low primary amino acid homology (LPH) between A1 and A3, at residues 268–400 [23]. Two earlier studies implicated the N-terminus (N) of A1, and residues 1–8 and 1–17 as necessary for A1 membrane localization [21,30]. While the earlier studied also implicated a C-terminal dileucine motif as contributing to the membrane localization of A, this was not as significant in comparison to N truncations [21]. Thus, we hypothesize both the N and a region of the LPH, the low homology domain, may be involved in A1 membrane association. In this study, A3-A1 N/LHD chimeras were used to study how N and the LHD could facilitate LC association with the plasma membrane. Steady-state and live imaging of the A3–A1 chimeras identified a two-step process where the N of light chain A bound intracellular vesicles, which facilitated an internal α-helical-rich domain (termed the low homology domain (LHD)) to mediate A plasma membrane association. Stable A plasma membrane association correlated with BoNT/A potency.

## 2. Results

### 2.1. Properties of A1 and A3

**Figure 1:**
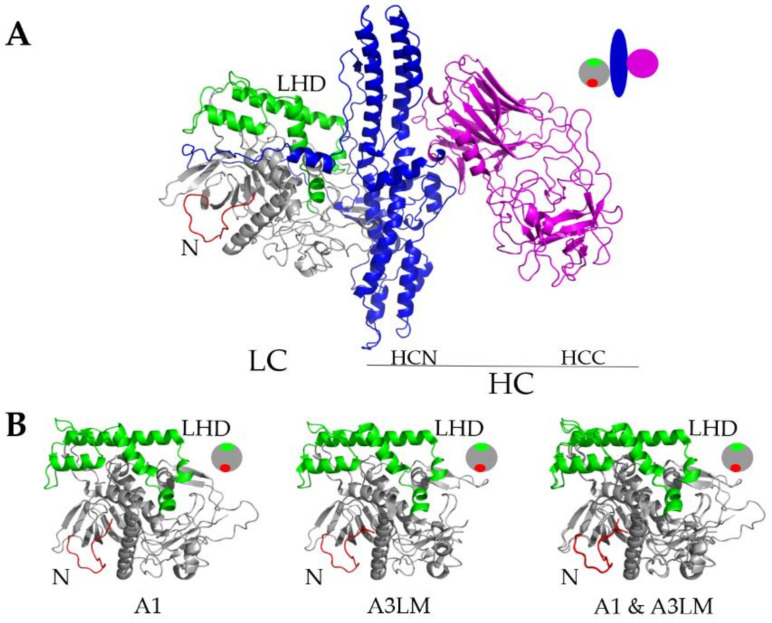
(**A**) Structure of botulinum neurotoxin A (BoNT/A) (PDB: 3BTA). (**B**) Structural alignments of A1 and A3LM. The light chain (LC) is shown in gray, the N in red, and LHD in green A1 (PDB: 1XTG) and middle A3LM (PDB: 7DVL) [31] and right A1 and A3LM merged with N (residues 1–17, red) and LHD (268–357, green) highlighted. Merge of A1 and A3LM was prepared with PyMol software. Circle schematic of LC:N (red), LHD (green), regions outside (gray), HC:HCN (blue), and HCC (magenta).

**Figure 2:**
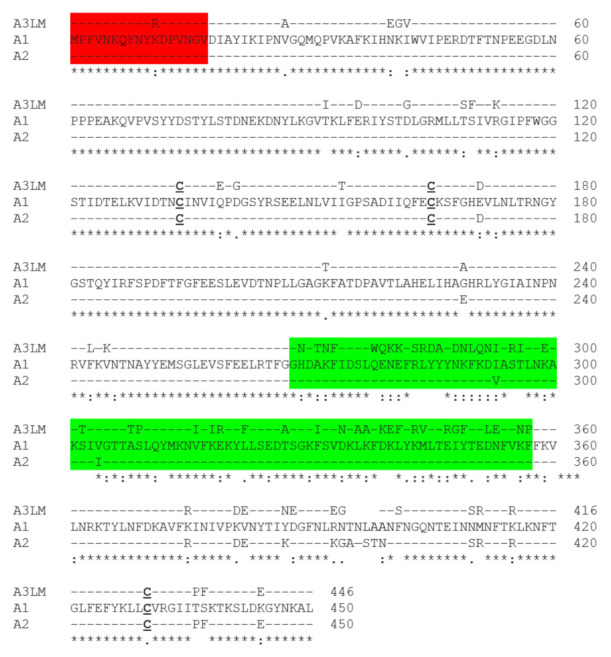
Blastp alignment of A3 versus A1. The primary amino acid sequences of A3LM (top) ACA57525, A1 (middle) ACS66881, and A2 (bottom) ADB85243 were analyzed by Blastp [32]. The bottom line depicts identical amino acids between A1, A2, and A3LM (_*_); conserved amino acids (:); and non-conserved amino acids ( ). Shown are the N region (amino acids 1–17, red) and the low homology domain (LHD, green), which comprises amino acids 268–357 and has ~60% identity between A3LM and A1. Cysteine residues are bolded and underlined.

The crystal structures of A1 and A3 strain Loch Maree (A3LM) had overall conserved secondary and tertiary structures (Figure 1). In addition, the A structures were conserved between the respective N-terminal 17 amino acids (N) and low homology domain (LHD) residues 268–357 [23,30]. These structural similarities implied that unique primary amino acid differences within the N and LHD were responsible for the differential duration and potency of BoNT/A1 and BoNT/A3, and for the differential intracellular localization of A1 and A3. Figure 2 highlights the primary amino acid sequences between A1, A3LM, and A2. Although outside the regions of interest, N, and LHD, there is a four-amino-acid deletion around the residue 400 present in A3LM. This deletion, found in an unstructured loop region, is not hypothesized to contribute to intracellular localization as this region is a connecting region to the higher homology C terminus. Within N, A1 and A2 were identical, while A3LM possessed a unique K^11^R substitution. Within the LHD, A1 and A2 were 97% identical, while A3LM had ~60% primary amino acid homology with A1 and A2, with differences in surface charge potential as there is a cluster of basic amino acids present in A3LM that are absent in A1 [31]. Supporting roles for the N and LHD in intracellular LC targeting, such as A1 ectopically expressing A2 at steady state, were localized to the plasma membrane (Supplemental Figure S1, see Supplementary Materials).

### 2.2. Variations in 17 N-Terminal Amino Acid Sequences (N) of BoNT/A3 Deposited in NCBI

Most BoNT/A3 sequences had 17 identical N sequences, except NCBI #ABY56337, which possessed two amino acid variations (Q^7^P and V^14^G), termed A3V, relative to A3LM (Figure 2). Upon further investigation, NCBI #ABY56337 is a subclone of A3LM, which, upon resequencing (NCBI #ACA57525), lacked the two additional point mutations (Q^7^P and V^14^G). Thus, the previously published A3V sequence containing Q^7^P and V^14^G in the N may be due to sequencing inaccuracy. Note, the primary amino acid sequences of the LHD of A3LM and A3V are identical. Subsequent determinations that A3LM and A3V possess different intracellular localizations when expressed ectopically in N2As provided a unique opportunity to characterize the basis for A intracellular location, as described below.

### 2.3. Unique Intracellular Localizations of A1, A3LM, and A3V in N2As

**Figure 3:**
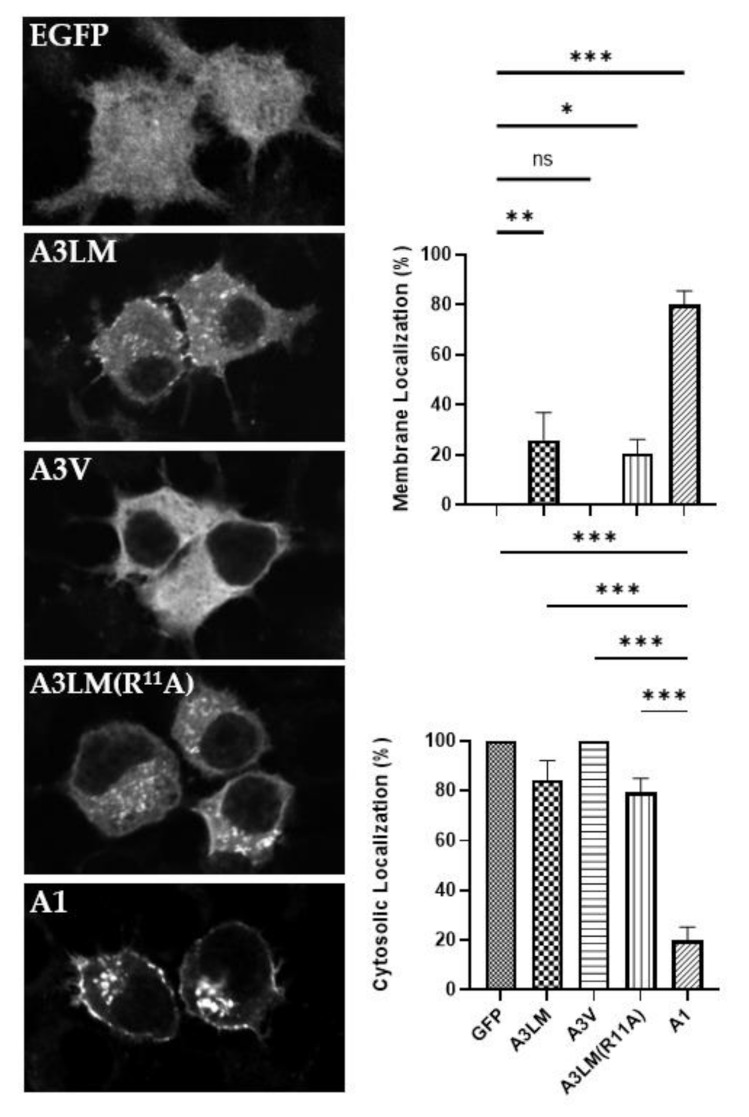
Intracellular localization of A3 N sequence variants. (Left) After overnight transfections, N2A cells were fixed with 4% paraformaldehyde and imaged for EGFP fluorescence (excitation 488 nm, emission 509 nm). Representative images show the steady-state localization of EGFP, EGFP-LC/A3 Loch Maree (A3LM), EGFP-LC/A3V (A3V), EGFP-LC/A3LM(R^11^A) (A3LM(R^11^A)), and EGFP-LC/A1 (A1). (Right) Percentage of EGFP membrane-bound or present in the cytosol. Ten random fields were selected and counted for membrane (Upper) or cytosolic (Lower) localization. Mean and SEM were evaluated, with ordinary one-way ANOVA with Dunnett’s multiple comparisons test using A3 LM as the control column: ns, not significant; * *p* < 0.01; ** *p* < 0.05; *** *p* < 0.001.

Steady-state ectopically expressed A1 localized on plasma membranes, with a fraction associated on intracellular vesicles, while A3LM localized in the cytosol and on intracellular vesicles, and A3V was expressed in the cytosol (Figure 3). In addition, A3LM(R^11^A) remained localized on intracellular vesicles, indicating that the presence of either R^11^ or K^11^ within the N did not influence intracellular localization, and implicated that Q^7^ and/or V^14^ are required for steady-state LC localization on intracellular vesicles. Other experiments showed that individual substitutions of either Q^7^P or V^14^G in A1 did not change the localization of the LC to cytosolic, indicating that both P^7^ and G^14^ are required to convert A1 to the cytosolic phenotype (Supplemental Figure S2). Overall, to the best of our knowledge, this is the first detection of A possessing an intracellular vesicle phenotype and implying both the N and LHD contribute to the intracellular localization of A1 to the plasma membrane and A3LM to intracellular vesicles, which were next tested. Additionally, GFP fusion to the N of A did not inhibit intracellular interactions, since individual point mutations and N interactions were not masked.

### 2.4. Contributions of A1-N and A1-LHD in Targeting A1 on the Plasma Membrane

**Figure 4:**
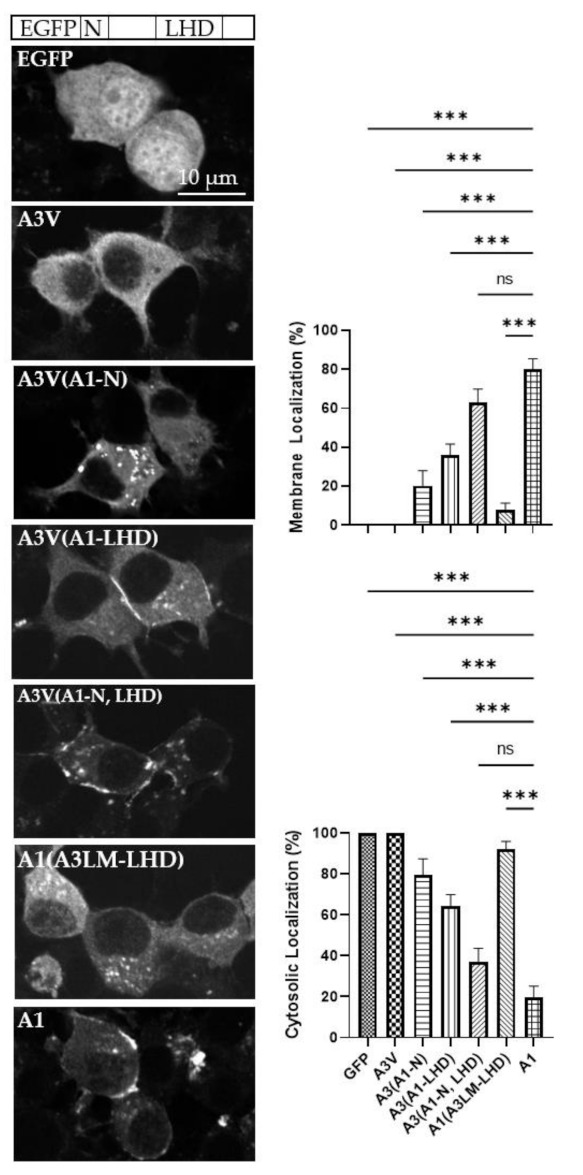
LC/A1 N and LHD are necessary and sufficient to localize A3V to the plasma membrane of N2A cells. After overnight transfection, N2A cells were fixed with 4% paraformaldehyde and imaged for EGFP fluorescence (excitation 488 nm and emission 509 nm). (Left) Schematic representation of the construct with the region of interest depicted as EGFP fluorescent tag, N terminus residues 1–17 (N), and low homology domain (LHD) residues 268–357. Representative images show the steady-state localization of EGFP, GFP-LC/A3V, EGFP-LC/A3V(A1-N), EGFP-LC/A3V(A1-LHD), EGFP- LC/A3V(A1-N, LHD), EGFP-LC/A1(A3LM-LHD), or EGFP-LC/A1 (A1). (Right) Percentage of EGFP membrane- or cytosol-localized. Ten random fields were selected and counted for membrane (upper) or cytosolic (lower) localization. Mean and SEM were evaluated, with ordinary one-way ANOVA with Dunnett’s multiple comparisons test using A1 as the control column: ns, not significant; *** *p* < 0.001.

The unique cytosolic location of A3V provided a tool to examine the basis of A1 intracellular localization, allowing the construction of A3V-A1 chimeras that contained A1-N, A1-LHD, or A1-N and A1-LHD engineered into the A3V backbone (Table 1). At steady state, A3V(A1-N), which replaced the N of A3V with the N of A1, possessed a similar phenotype to A3LM, being cytosolic and localized on intracellular vesicles (Figure 4). This showed that the N is responsible for LC localization to intracellular vesicles. Next, A3V(A1-LHD), which replaced the LHD of A3V with the LHD of A1, was partially localized on the plasma membrane with fractional presence in the cytosol (Figure 4). This phenotype, which indicated that while the A1-LHD is sufficient for A transition to the plasma membrane, the ability to interact with intracellular vesicles is primarily but not solely mediated by the N. Next, A3V(A1-N, LHD), which replaced the N and LHD of A3V with the N and LHD of A1, was primarily localized on the plasma membrane with a statistically significant amount LC bound to intracellular vesicles, as with A1 (Figure 4). Overall, the long-term steady-state experiments showed that A1-N and A1-LHD are necessary and sufficient to efficiently transition A3V from the cytosol to the plasma membrane.

In a reciprocal experiment, A1(A3LM-LHD), which replaced the LHD of A1 with the LHD of A3LM, localized on intracellular vesicles with some LC present in the cytosol, but without steady-state localization on the plasma membrane, which further confirmed that A1-LHD is required for stable A association with the plasma membrane (Figure 3).

### 2.5. The LHD of A Defines Affinity for the Plasma Membrane

Steady-state analysis of various As and A3V-A1 chimeras implicated a role for N association with intracellular particles (Figure 3) and LHD for an association with the plasma membrane (Figure 4), respectively.

**Figure 5:**
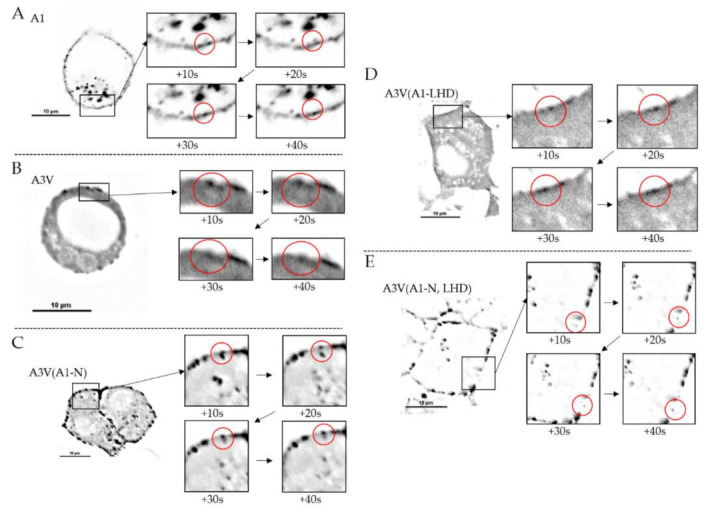
Time-lapse imaging of EGFP-LC/A. After a five-hour transfection of the indicated EGFP-LC/A (**A**); EGFP-LC/A1 (A1) (**B**) EGFP-LC/A3V (A3V) (**C**); EGFP-LC/A3V(A1-N), (A3V(A1-N)) (**D**); and EGFP-LC/A3V(A1-LHD) (A3V(A1-LHD)) (**E**). EGFP-LC/A3V(A1-N, LHD) (A3V(A1-N, LHD)) and N2As were imaged on a Nikon Eclipse Ti2 microscope equipped with a W1 Spinning Disc, Orca Flash CMOS camera, and 60× oil-immersion objective (CFI Plan Apo λ, 1.4 NA objective) confocal microscope [33]. Live-cell images were obtained every 10 s for 10 min. Images were deconvoluted with Nikon Elements Deconvolution Software (Version 6).

Time-lapse imaging showed A1 was present in a vesicle and these vesicles possessed anterograde movement to the plasma membrane, and that A1 was retained in the plasma membrane over the 10 min observation period. This indicated that A1 mediates a stable interaction with the plasma membrane (Figure 5A). As a control, A3V was examined for intracellular localization, knowing that at steady state, A3V is expressed as a cytosolic protein (Figure 3). However, early EGFP fluorescence was detected in the cytosol and occasionally on the plasma membrane, but fluorescence did not persist on the plasma membrane, which indicated that the plasma-membrane-bound A3V returns to the cytosol (Figure 5B). Next, the roles of A1-N and A1-LHD in transitioning A3V from the cytosol to intracellular vesicles and trafficking to the plasma membrane were investigated. By time-lapsed imaging, A3V(A1-N), which replaced the N terminus, residues 1–17 of A3V with A1-N, demonstrated bi-directional movement of A3V(A1-N) to the plasma membrane, with detectable association and dissociation of A3V(A1-N) with the plasma membrane (Figure 5C). This is consistent with the observed detection of A3V(A1-N) on intracellular vesicles in the steady-state analysis (Figure 4). This also indicated that A3-LHD mediates reversible A interactions with the plasma membrane. By time-lapsed imaging, A3V(A1-LHD), which replaced the A3-LHD and the A1-LHD, fluorescence was first detected in the cytosol, followed by a partial accumulation on the plasma membrane (Figure 5D). This indicated A1-LHD directs A movement from the cytosol to the plasma membrane, which retains A on the plasma membrane. Finally, by time-lapsed imaging, A3V(A1-N, LHD), which replaced the N and LHD of A3V with the N and LHD of A1, respectively, fluorescence detected intracellular vesicles with significant accumulation on the plasma membrane (Figure 5E), indicating LC trafficking to and retention on the plasma membrane. Imaging of A3V(A1-N, LHD) was similar to the time-lapse imaging of A1. Thus, A1-N localized A to intracellular vesicles and A1-LHD is necessary for A accumulation on the plasma membrane. Together, A1-N and A1-LHD are necessary and sufficient to convert A3V from a cytosolic protein to a plasma-membrane-localized protein similar to A1.

### 2.6. Association with Plasma Membrane Correlates with BoNT/A Potency

As the additional two amino acid mutations of the N region of A3V resulted in the cytosolic distribution versus the mixed cytosolic and intracellular vesicle distribution of A3LM, the role of the N variations in BoNT potency was tested relative to BoNT/A1. Recombinant BoNT/A3V holotoxin was engineered and purified from *C. botulinum* strain Hall A hyper tox-. Purified rBoNT/A3V migrated as a 150 kDa single-chain protein by non-reduced SDS-PAGE, and 100 and 50 kDa dichain protein in reduced SDS-PAGE, indicating that rBoNT/A3V was >90% dichain protein (Supplemental Figure S3). In a mouse bioassay for botulism, purified rBoNT/A3V possessed a specific activity of 1 × 10^7^ LD_50_/mg (100 pg/LD_50_) relative to BoNT/A3LM, which possessed a specific activity of 5.8 × 10^7^ LD_50_/mg (17 pg/LD_50_); and BoNT/A1, which possessed a 1–2 × 10^8^ LD_50_/mg (5–10 pg/LD_50_) (Table 2) [34]. An ongoing studying is being performed to investigate if the intracellular localization of A3LM and A3V contributes to the duration of action. These data showed that BoNT/A3V is 6-fold less potent than BoNT/A3LM, and 10–20-fold less potent than BoNT/A1, showing that the efficiency of intracellular A distribution to the plasma membrane correlates with BoNT/A potency.

## 3. Discussion

**Figure 6:**
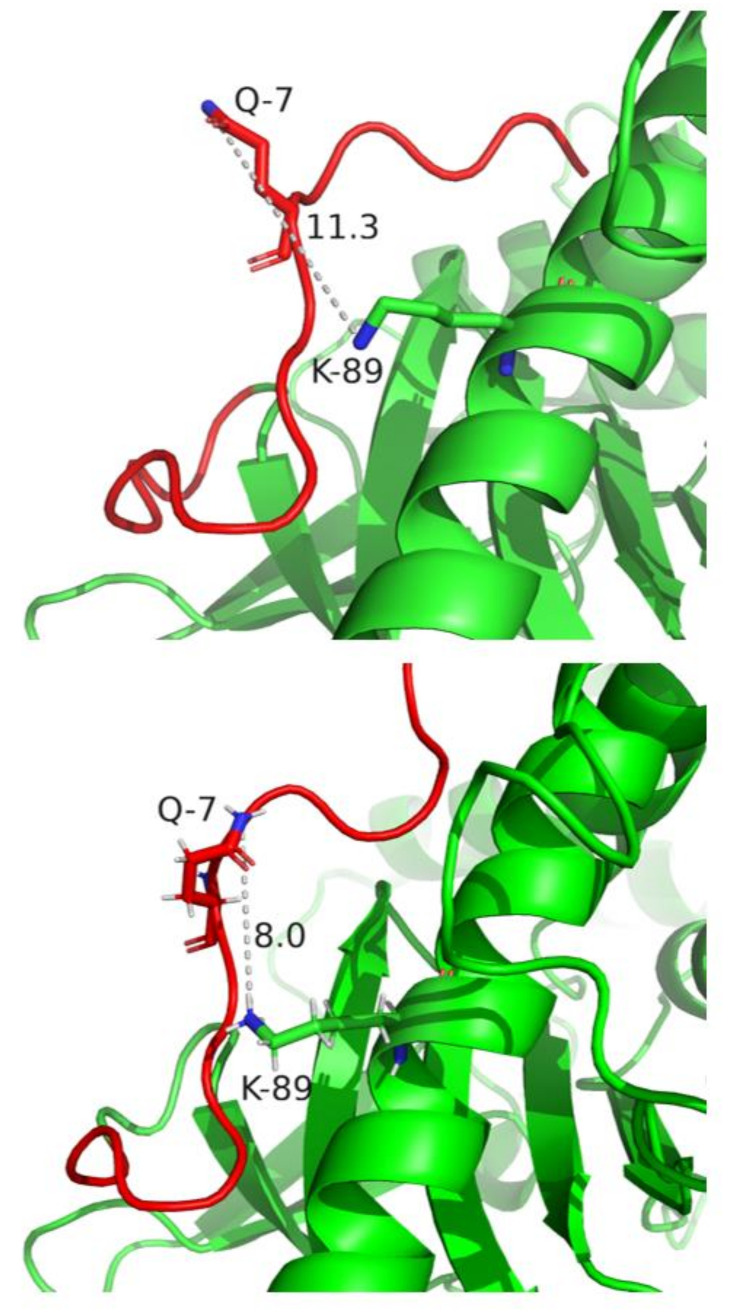
Structures of the proposed A1 and A3(LM) intramolecular interactions. (**Top**) Crystal structure of A1 (PDB:1XTG) and (**Bottom**) A3LM (PDB:7DVL) distance between glutamine (Q^7^) and lysine (K^89^) as measured with PyMOL. The N of A1 and A3LM is highlighted in red with the remaining regions of LC in green.

While the unique amino acid compositions of the N and LHD (Figure 2) correlated to A1 plasma membrane association and A3 cytosolic presence [23,30], how the N and LHD contributed to A intracellular location was not known. In the current study, we used a cultured neuron cell localization assay to study the transition of A3V from a cytosolic protein to a vesicle- and/or plasma-membrane-bound protein with the addition of the N and LHD of A1. Knowing how the unstructured A1 N folds into a pocket within the groove of the LC may provide insight into the molecular interactions responsible for the role of the N in vesicle association. For example, analysis of the primary amino acid sequences between A1 and A3 showed glutamine in the seventh position of A1 and A3LM and proline in the same position in A3V, which may interact with lysine^89^ in a closely juxtaposed α helix. Examination of the A1 (PDB:1XTG) and A3 (PDB:7DVL) crystal structures showed a possible Q^7^-K^89^ interaction. While the crystal structure of A1 showed an 11.3 Å distance between these two residues, the crystal structure of A3LM showed an 8.0 Å between Q^7^ and K^89^, implying that the fluidity of this unstructured region may allow Q^7^ to form a noncovalent interaction with K^89^ (Figure 6). This proposed noncovalent interaction may hold the unstructured N region of A1 and A3LM, but not A3V, which possesses P^7^, in a conformation that allows intracellular interactions that contribute to vesicle association. While the nature of the observed intracellular vesicles is unknown, these particles may be SV-like vesicles, since earlier studies of N2As showed HCC/A1 colocalized with SV2C-positive intracellular vesicles [36]. The role of the LC-N domain for vesicle association and membrane trafficking is reminiscent of ExoS, a type III cytotoxin of *Pseudomonas aeruginosa*, which utilizes an N-terminal domain (residues 51–77) to localize to intracellular vesicles. Removal of the ExoS N, akin to A1(Δ1–17), transitioned ExoS to the cytosol, showing that although structurally different, both the N of A1 and the N of ExoS are functionally similar [37]. Understanding how other protein toxins utilize N-like regions for intracellular localization and if these regions correlate to potency may help define early steps in the intracellular trafficking of protein toxins to their intracellular targets. Current studies are addressing BoNT/A3V’s duration of action; while still ongoing, we hypothesize that similar to data already observed, the fully cytosolic A3V will have a shorter duration than A3LM due to ubiquitination and degradation by the proteasome, since proteins that are membrane-bound or in a protein complex have a longer half-life than the same protein found in the cytosol [38]. Understanding how A localizes to the plasma membrane may provide insight into mechanisms controlling potency to extend BoNT/A1 as a human therapy.

The exchange of A1-LHD to the respective region of A3V transitioned the intracellular A3(A1-LHD) localization from soluble in the cytosol to partially plasma-membrane-associated, indicating that A1-LHD has an intrinsic affinity for the plasma membrane that appears independent of the association with intracellular vesicles (Figure 4). This was further resolved by live imaging, which revealed that A1-LHD contributed to increasing transition and sequestration to the plasma membrane from the cytosol over time (Figure 5). Regional mapping of the crystal structure, SNAP-25 bound to A1 (PDB:1XTG), showed that SNAP-25-A1 interacting residues are external of the LHD, indicating that the LHD may not play a direct role in the cleavage of SNAP-25 [33]. However, within the LHD, there are three α helices and two loop regions (Figure 1). Residues 275–300 in A1 are a long surface exposed to an α helix, which has 36% homology with A3. Of the 16 residues that differ within this α helix, 15 are surface-exposed, possibly leading to this region having external interactions. Additionally, this α helix has amphipathic characteristics at the C-terminal end, which may give rise to lipid interactions. The other region within the LHD, residues 335–357, is a helical bundle composed of two α helixes separated by a short two amino acid unstructured region, a helix-turn-helix motif. This helical bundle is juxtaposed to SNAP-25, although previous mapping models showed this region within the LHD does not interact with SNAP-25 residues 141–206, there could be electrostatic interactions that stabilize the transition from the vesicle to the association with SNAP-25 [30]. These two regions, residues 275–300 and 335–357, show that the LHD could possess internal interactions with SNAP-25 or external interactions with the plasma membrane and/or unknown protein(s), leading to the membrane phenotype. The A2 membrane localization also supports a role of the LHD in membrane localization since A2 is 97% homologous in the LHD to A1. The exchange of A1-N and A1-LHD transitioned A3V to an A1 phenotype, indicating A1-N and A1-LHD are necessary and sufficient to target A3V from the cytosol to intracellular vesicles that moved in an anterograde fashion to the plasma membrane (Figure 4).

**Figure 7:**
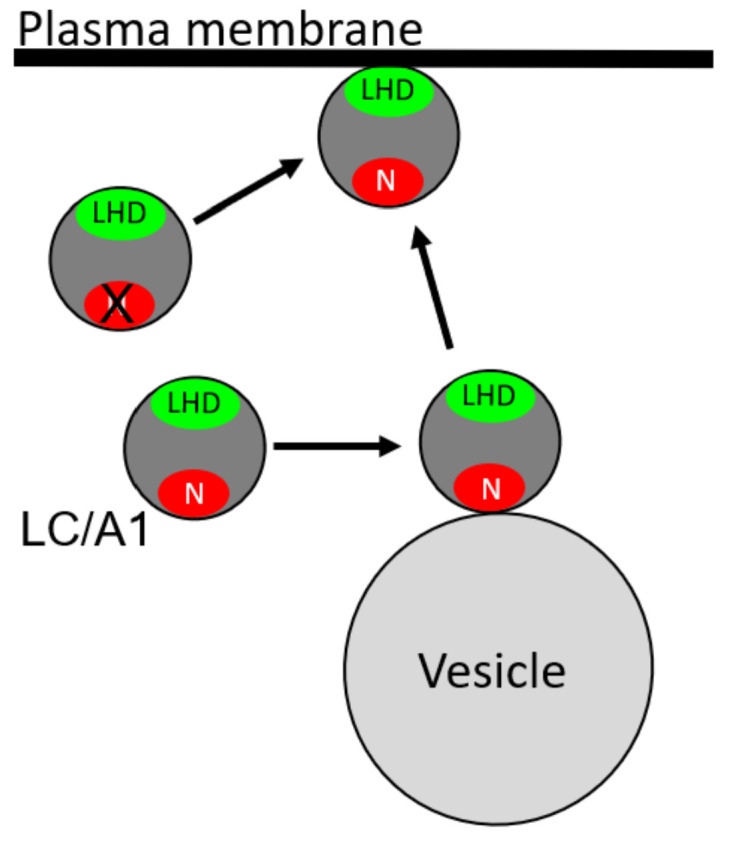
Model for the anterograde trafficking and localization of A1 to the plasma membrane. The N (red) of A1 associates with the extracellular surface of the vesicle; the LHD (green) allows A1 to efficiently associate with the plasma membrane. LHD transitions both soluble and vesicular-bound A1 to the plasma membrane independent of the N of A1, but transfers are more efficient when LC is vesicle-associated.

Our findings showed that the N and LHD of A1 contribute sequentially toward the intracellular association of A1 to the plasma membrane. While previously published works proposed a C-terminal dileucine motif contributing to A membrane localization, our data indicate that this region is not necessary for intracellular trafficking [21]. An extrapolation of these data allowed the construction of a model for the A1-N and A1-LHD membrane interactions (Figure 7). First, at steady state, A3V is cytosolic, which predicts that after A translocation out of the lumen of a synaptic vesicle, A3V diffuses into the cytosol. Second, A3LM and A1 localize to unique intracellular vesicles, indicating A1-N or A3LM-N allow A to associate with an intracellular vesicle and, since anterograde trafficking of vesicles to the membrane can occur, a partial membrane-associated phenotype is observed. Third, A3V(A1-LHD) distributed between the cytosolic and plasma membrane, indicating that A1-LHD is necessary and sufficient for association with the plasma membrane, although less efficient than A1 due to a lack of directed trafficking mediated by A1-N. Additionally, intracellular localization to the plasma membrane correlated with the potency of BoNT/A1, as the potency of BoNT/A3V (cytosolic) was lower than that of BoNT/A3LM (primarily intracellular-vesicle-localized and partially with the plasma membrane), which, in turn, is lower than that of BoNT/A1, which is primarily associated with the plasma membrane.

## 4. Materials and Methods

Reagents were purchased from Life Technologies (Grand Island, NY, USA) unless otherwise specified.

### 4.1. Sequence and Structural Alignment

Primary amino acid sequences for BoNT/A1 (ACS66881), BoNT/A2 (ADB85243), and BoNT/A3LM (ACA57525) were obtained from the Uniprot [39] and NCBI databases [40]. Protein sequence alignment was performed with the Blastp Suite (U.S. Nation Library of Medicine) [32] to identify variations between the N (amino acids 1–17) [30] and low primary amino acid homology of A1 (258–400) and A3 (268–396) [23]. Examining these regions, amino acids 268–357 were identified as the longest contiguous region having low primary amino acid homology between A1 and A3 (54%) (termed the LHD), which was used in the current study (Figure 2).

### 4.2. Construction of LC/A and LC/A-Chimera Expression Plasmids 

DNA encoding A1 (1–450) and A3 (1–446) was engineered as green fluorescent protein (EGFP) fusions, GFP-small linker-A, subcloning the LC genes into the SacI-BamHI restriction sites of pEGFP-C3. New England Biolabs^®^ NEBaseChanger^®^ (Ipswich, MA, USA) was used for primer design to engineer genes encoding several EGFP-A3-A1 chimeras: A3(A1 1–17), A3(A1 268–357), and A3(A1 1–17, A1 268–357) (Table 1). Point-mutated EGFP-LC/A3 variants were also engineered, including A3, A3(R11K), A3(R11A), and A3(Q^7^P and V^14^G).

We performed expression and scoring of steady-state intracellular localization of EGFP-LC/A fusion proteins in neuro-2A cells (N2As, ATCC (Manassas, VA, USA) CCL-131). N2As were plated as previously described [19]. The next day, N2As were transfected (Lipofectamine LTX; Invitrogen™ (Waltham, MA, USA)) with 0.5 µg of the indicated plasmid, as described by the manufacturer. Following overnight incubation, N2As were fixed with 4% paraformaldehyde and incubated for 30 min at 4 °C with wheat germ agglutinin:Alexa Fluor^647^(1:1000) as a membrane marker and DAPI as a nuclear marker, and imaged with a Nikon Eclipse Ti-inverted microscope, using a 60× 1.4 NA objective and Eclipse software for data analysis. Ten random fields of N2As from each transfection were scored for EGFP localization (excitation 488 nm and emission 509 nm), scoring a total of ~100 cells. EGFP was scored positive for membrane-localized when EGFP colocalized with wheat germ agglutinin, and was scored positive for cytosol localization when the EGFP signal was detected in the cytosol. Only the transfected pEGFP vector localized to the nucleus with DAPI. Positive cells were scored as EGFP-localized on the membrane or in the cytosol/(total number of EGFP-positive cells) × 100 [23]. Results were graphed with GraphPad Prism 9 (San Diego, CA, USA) and subjected to a statistical test. Statistical significance was tested using ordinary one-way ANOVA with Dunnett’s multiple comparisons test with A1 as the control column. Western blotting showed that each EGFP-A fusion protein was expressed at similar levels and migrated with the anticipated molecular weight by SDS-PAGE (Supplemental Figure S4).

### 4.3. Live-Cell Imaging 

Cells were plated as previously described [19]. Five hours post-transfection, live cells were imaged on a Nikon Eclipse Ti2 microscope equipped with a W1 Spinning Disc, Orca Flash CMOS camera, and 60× oil-immersion objective (CFI Plan Apo λ, 1.4 NA objective) confocal microscope, as previously described [41]. Live-cell images were obtained every 10 seconds. Videos and obtained images were deconvoluted utilizing Nikon Elements deconvolution software version 6 (Melville, NY, USA).

### 4.4. Generation of a Recombinant Gene for Expression of BoNT/A3V 

The BoNT/A3 gene was amplified by PCR using the total genomic DNA isolated from *C. botulinum* subtype A3 strain (kindly provided by the CDC (Atlanta, GA, USA) and Fusion Hot Start Flex 2× Master mix according to the manufacturer’s instructions (New England Biolabs). To introduce two amino acid mutations into the N terminus of the LC/A, two nucleotide substitutions were included into the 5′ PCR primer: mutation Gln^7^ to Pro (CAA to C**C**A) and Val^14^ to Gly (GTA to G**G**A). Primers A3-Nde-5′ and A3-Sal-3′ were used to amplify the *bont/A3V* gene.

A3-Nde-5′ GGCATATGCCATTTGTTAATAAAC**C**ATTTAATTATAGAGATCCTG**G**AAATGGTG

A3-SalI-3′ GCGTCGACCTTACAGTGAACTTTCTCCCCATCCATCATC

The nucleotide sequence of the mutated *bont/A3V* was verified by DNA sequencing. Then, the recombinant gene was inserted into modular clostridial expression vectors pMTL82152 and pMTL83152 [42]. Recombinant expression vectors were transferred into nontoxigenic *C. botulinum* expression host strain Hall A-*hyper*/tox^-^ by conjugation from *E. coli* donor strain CA434 and expressed as previously described [43].

### 4.5. Production of BoNT/A3V 

Recombinant BoNT/A3V was expressed in the atoxic *C. botulinum* strain Hall A-*hyper*/tox^-^ [43] using the pMTL82152 vector [42]. rBoNT/A3V was purified according to the method previously described for the purification of BoNT/A1 [44], with an additional chromatography step after the DEAE, pH 8.0 column to remove complex proteins on a 1.5 mL p-aminobenzyl 1-thio-b-D- galactopyranoside (pABTG) agarose affinity column, as previously described [45]. Purified rBoNT/A3V(Q^7^P, V^14^G)toxin was passed through a 0.2 µm filter and mixed with an equal volume of 80% sterile glycerol and stored at –20 °C. Purified rBoNT/A3V (rA3V) was analyzed by 4–12% Bis-Tris (Novex NuPAGE, Life Technologies) gel electrophoresis either nonreduced or reduced with 100 mM DTT (Sigma-Aldrich, St Louis, MO, USA) and stained with InstantBlue (Abcam, Cambridge, MA, USA). Concentration and purity were determined by spectroscopy (absorbance at 278 nm) and densitometry.

### 4.6. Mouse Bioassay 

The activity of rBoNT/A3V was determined using a standard intraperitoneal mouse bioassay (MBA) as previously described [43,45]. Briefly, rBoNT/A3V was diluted to a final concentration of 44, 88, 134, 178, 266, and 356 pg/mL in GelPhosphate buffer (0.03 M sodium phosphate, pH 6.3, 0.2% gelatin (Sigma-Aldrich)). Groups of 4 female ICR mice (18–22 g) (Envigo, Madison, WI, USA) were injected intraperitoneally (i.p.) with 0.5 mL of each dilution and observed for 4 days for signs of botulism and death. The specific activity was calculated according to the method of Reed and Muench [35].

### 4.7. Western Blot 

Twenty-four hours post-transfection with DNA encoding the indicated plasmid, N2As were lysed in 2× protein sample buffer (150 µL). Cell lysates were subjected to SDS-PAGE, transferred to Immobilon-P polyvinylidene difluoride membranes (Millipore, Billerica, MA, USA); after a 60 min, incubation in blocking solution (2% in 0.1% TBST) membranes were incubated with rat α-EGFP-monoclonal IgG (3H9, Chromotek, Planegg, DE, 1:5000 final dilution) or mouse α-β-actin monoclonal IgG (A2228, Millipore Sigma, Mass, USA, 1:20,000 final dilution). Bound primary antibodies were recognized as α-rat or α-mouse IgG secondary antibody conjugated with horseradish peroxidase (Life Technologies, MA, USA, 1:1000), and secondary antibodies were visualized using Super Signal™ West Pico PLUS Chemiluminescent Substrate (34578, Thermo, IL, USA) on an Azure C600 Imaging System (Dublin, CA, USA), using a 60 s exposure.

### 4.8. Structural Alignment 

Crystal structures of A1 (PDB:1XTG) and A3LM (PDB:7DVL) were analyzed using PyMOL (PyMOL Molecular Graphic System, Version 2.0 Schrödinger, LLC. (New York, NY, USA)). The crystal structures of both proteins were aligned to one another, and regions of interest were highlighted in red or green. Structures were ray traced using Ray command and images exported as a PNG.

## 5. Conclusions

This study characterized two regions within LC/A, N and LHD, that contribute sequentially to the LC/A1 membrane-associated phenotype. Although LC/A3V may have been detected based on a sequencing error, these studies implicate LC/A3V as a platform to identify additional steps in the intracellular trafficking of LC/A1 and LC/A3LM, an approach that can be adapted for identifying LC function in other BoNT serotypes. Understanding the cellular basis of LC intracellular localization provides insight to BoNT/A potency for extensions to applications as a human therapy.

## Figures and Tables

**Table 1:** EGFP-LC/A3-A1 chimeras analyzed in this study.

LC/A3-A1 Chimeras ^1^	Designation
LC/A1	A1
LC/A3V(A1 1–17)	A3V(A1-N)
LC/A3V(A1 268–357)	A3V(A1-LHD)
LC/A3V(A1 1–17, A1 268–357)	A3V(A1-N, LHD)

^1^ Chimeras contained LC/A3V platform with the indicated exchange of A1 region N and/or LHD.

**Table 2:** Potency of BoNT/A variants in a mouse model of botulism ^1^.

**Light chain localization**	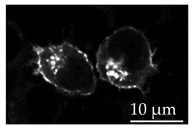	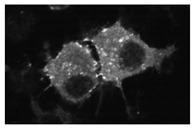	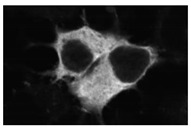
**BoNT variant**	BoNT/A1	BoNT/A3LM	BoNT/A3V
**Potency pg/LD_50_**	5–10 pg/LD_50_	17 pg/LD_50_	100 pg/LD_50_
**References**	[28,34]	[34]	this study

^1^ Groups of 4 female ICR mice (18–22 g) were injected intraperitoneally with 0.5 mL of each BoNT dilution and observed for 4 days for signs of botulism and fatality. Specific activity was calculated according to the method of Reed and Muench [35].

## Supplementary Materials

Supplemental Figures S1–S4 are available online at https://www.mdpi.com/article/10.3390/ijms222011115/s1.

## References

[1] Schiavo G., Matteoli M., Montecucco C. (2000). Neurotoxins Affecting Neuroexocytosis. Physiol. Rev..

[2] Johnson E.A., Bradshaw M. (2001). Clostridium botulinum and its neurotoxins: A metabolic and cellular perspective. Toxicon.

[3] Aoki K.R., Guyer B. (2001). Botulinum toxin type A and other botulinum toxin serotypes: A comparative review of biochemical and pharmacological actions. Eur. J. Neurol..

[4] Singh B.R. (2002). Scientific and Therapeutic Aspects of Botulinum Toxic.

[5] Singh B.R., DasGupta B. (1990). Conformational changes associated with the nicking and activation of botulinum neurotoxin type E. Biophys. Chem..

[6] Schiavo G., Rossetto O., Santucci A., Dasgupta B.R., Montecucco C. (1992). Botulinum neurotoxins are zinc proteins. J. Biol. Chem..

[7] Südhof T.C., Rothman J.E. (2009). Membrane Fusion: Grappling with SNARE and SM Proteins. Science.

[8] Binz T., Sikorra S., Mahrhold S. (2010). Clostridial Neurotoxins: Mechanism of SNARE Cleavage and Outlook on Potential Substrate Specificity Reengineering. Toxins.

[9] Henkel J.S., Baldwin M.R., Barbieri J.T. (2010). Toxins from bacteria. Exp. Suppl..

[10] Brunger A.T., A Breidenbach M., Jin R., Fischer A., Santos J.S., Montal M. (2007). Botulinum Neurotoxin Heavy Chain Belt as an Intramolecular Chaperone for the Light Chain. PLOS Pathog..

[11] Chen S., Kim J.-J.P., Barbieri J.T. (2007). Mechanism of Substrate Recognition by Botulinum Neurotoxin Serotype, A.J. Biol. Chem..

[12] Peck M.W., Smith T.J., Anniballi F., Austin J.W., Bano L., Bradshaw M., Cuervo P., Cheng L.W., Derman Y., Dorner B.G. (2017). Historical Perspectives and Guidelines for Botulinum Neurotoxin Subtype Nomenclature. Toxins.

[13] Dong M., Yeh F., Tepp W.H., Dean C., Johnson E.A., Janz R., Chapman E.R. (2006). SV2 Is the Protein Receptor for Botulinum Neurotoxin A. Science.

[14] Schengrund C.-L., Dasgupta B.R., Ringler N.J. (1991). Binding of Botulinum and Tetanus Neurotoxins to Ganglioside GT1b and Derivatives Thereof. J. Neurochem..

[15] Rummel A. (2012). Double Receptor Anchorage of Botulinum Neurotoxins Accounts for their Exquisite Neurospecificity. Curr. Top. Microbiol. Immunol..

[16] Pirazzini M., Rossetto O., Eleopra R., Montecucco C. (2017). Botulinum Neurotoxins: Biology, Pharmacology, and Toxicology. Pharmacol. Rev..

[17] Hoch D.H., Romero-Mira M., Ehrlich B., Finkelstein A., DasGupta B.R., Simpson L.L. (1985). Channels formed by botulinum, tetanus, and diphtheria toxins in planar lipid bilayers: Relevance to translocation of proteins across membranes. Proc. Natl. Acad. Sci. USA.

[18] Falnes P.Ø., Olsnes S. (1995). Cell-mediated Reduction and Incomplete Membrane Translocation of Diphtheria Toxin Mutants with Internal Disulfides in the A Fragment. J. Biol. Chem..

[19] Zuverink M., Chen C., Przedpelski A., Blum F.C., Barbieri J.T. (2015). A Heterologous Reporter Defines the Role of the Tetanus Toxin Interchain Disulfide in Light-Chain Translocation. Infect. Immun..

[20] Tehran D.A., Pirazzini M., Leka O., Mattarei A., Lista F., Binz T., Rossetto O., Montecucco C. (2017). Hsp90 is involved in the entry of clostridial neurotoxins into the cytosol of nerve terminals. Cell. Microbiol..

[21] Fernandez-Salas E., Steward L.E., Ho H., Garay P.E., Sun S.W., Gilmore M.A., Ordas J.V., Wang J., Francis J., Aoki K.R. (2004). Plasma membrane localization signals in the light chain of botulinum neurotoxin. Proc. Natl. Acad. Sci. USA.

[22] Schiavo G., Rossetto O., Catsicas S., De Laureto P.P., Dasgupta B.R., Benfenati F., Montecucco C. (1993). Identification of the nerve terminal targets of botulinum neurotoxin serotypes A, D, and E. J. Biol. Chem..

[23] Pellett S., Bradshaw M., Tepp W.H., Pier C.L., Whitemarsh R.C.M., Chen C., Barbieri J.T., Johnson E.A. (2018). The Light Chain Defines the Duration of Action of Botulinum Toxin Serotype A Subtypes. mBio.

[24] Jacky B.P.S., Garay P.E., Dupuy J., Nelson J.B., Cai B., Molina Y., Wang J., Steward L.E., Broide R.S., Francis J. (2013). Identification of Fibroblast Growth Factor Receptor 3 (FGFR3) as a Protein Receptor for Botulinum Neurotoxin Serotype A (BoNT/A). PLOS Pathog..

[25] Kull S., Schulz K.M., Strotmeier J.W.N., Kirchner S., Schreiber T., Bollenbach A., Dabrowski P.W., Nitsche A., Kalb S., Dorner M.B. (2015). Isolation and Functional Characterization of the Novel Clostridium botulinum Neurotoxin A8 Subtype. PLoS ONE.

[26] Smith T.J., Lou J., Geren I.N., Forsyth C.M., Tsai R., LaPorte S.L., Tepp W.H., Bradshaw M., Johnson E.A., Smith L.A. (2005). Sequence Variation within Botulinum Neurotoxin Serotypes Impacts Antibody Binding and Neutralization. Infect. Immun..

[27] Jacobson M.J., Lin G., Tepp W., Dupuy J., Stenmark P., Stevens R.C., Johnson E.A. (2011). Purification, Modeling, and Analysis of Botulinum Neurotoxin Subtype A5 (BoNT/A5) from Clostridium botulinum Strain A661222. Appl. Environ. Microbiol..

[28] Whitemarsh R.C.M., Tepp W.H., Johnson E.A., Pellett S. (2014). Persistence of Botulinum Neurotoxin A Subtypes 1-5 in Primary Rat Spinal Cord Cells. PLoS ONE.

[29] Pellett S., Tepp W.H., Whitemarsh R.C., Bradshaw M., Johnson E.A. (2015). In vivo onset and duration of action varies for botulinum neurotoxin A subtypes 1-5. Toxicon.

[30] Chen S., Barbieri J.T. (2011). Association of Botulinum Neurotoxin Serotype A Light Chain with Plasma Membrane-bound SNAP-25. J. Biol. Chem..

[31] Leka O., Wu Y., Li X., Kammerer R.A. (2021). Crystal structure of the catalytic domain of botulinum neurotoxin subtype A3. J. Biol. Chem..

[32] Shiryev S.A., Papadopoulos J.S., Schäffer A.A., Agarwala R. (2007). Improved BLAST searches using longer words for protein seeding. Bioinformatics.

[33] Breidenbach M.A., Brunger A. (2004). Substrate recognition strategy for botulinum neurotoxin serotype A. Nat. Cell Biol..

[34] Tepp W.H., Lin G., Johnson E.A. (2012). Purification and Characterization of a Novel Subtype A3 Botulinum Neurotoxin. Appl. Environ. Microbiol..

[35] Reed L., Muench H. (1938). A simple method of estimating fifty percent endpoints. Am. J. Hyg..

[36] Blum F.C., Chen C., Kroken A., Barbieri J.T. (2012). Tetanus Toxin and Botulinum Toxin A Utilize Unique Mechanisms to Enter Neurons of the Central Nervous System. Infect. Immun..

[37] Zhang Y., Deng Q., Barbieri J.T. (2007). Intracellular Localization of Type III-delivered Pseudomonas ExoS with Endosome Vesicles. J. Biol. Chem..

[38] Heo S., Diering G.H., Na C.H., Nirujogi R.S., Bachman J.L., Pandey A., Huganir R.L. (2018). Identification of long-lived synaptic proteins by proteomic analysis of synaptosome protein turnover. Proc. Natl. Acad. Sci. USA.

[39] The UniProt Consortium (2021). UniProt: The universal protein knowledgebase in 2021. Nucleic Acids Res..

[40] The UniProt Consortium (2019). UniProt: A worldwide hub of protein knowledge. Nucleic Acids Res..

[41] Dirck A.T., Whyte M., Hudson A.W. (2020). HHV-7 U21 exploits Golgi quality control carriers to reroute class I MHC molecules to lysosomes. Mol. Biol. Cell.

[42] Heap J.T., Pennington O.J., Cartman S.T., Minton N.P. (2009). A modular system for Clostridium shuttle plasmids. J. Microbiol. Methods.

[43] Bradshaw M., Tepp W.H., Whitemarsh R.C.M., Pellett S., Johnson E.A. (2014). Holotoxin Activity of Botulinum Neurotoxin Subtype A4 Originating from a Nontoxigenic Clostridium botulinum Expression System. Appl. Environ. Microbiol..

[44] Malizio C.J., Goodnough M.C., Johnson E.A. (2000). Purification of Clostridium botulinum Type A Neurotoxin. Bacterial Toxins.

[45] Moberg L.J., Sugiyama H. (1978). Affinity chromatography purification of type A botulinum neurotoxin from crystalline toxic complex. Appl. Environ. Microbiol..

