# Supplementary material for: Resolution of Two Steps in Botulinum Neurotoxin Serotype A1 Light Chain Localization to the Intracellular Plasma Membrane
Source: Int J Mol Sci. 2021 Oct 15;22(20):11115. doi: 10.3390/ijms222011115 (PMC8539409; doi:10.3390/ijms222011115)
Supplement: Supplementary file 1 [file ijms-22-11115-s001.zip › ijms-1396885-supplementary.pdf]

Authors: Alexander Gardner et al.,

Title: Resolution of two steps in Botulinum Neurotoxin serotype A1 Light Chain trafficking to the intracellular plasma membrane.

Topic: Suppelmenal Figures 1-4

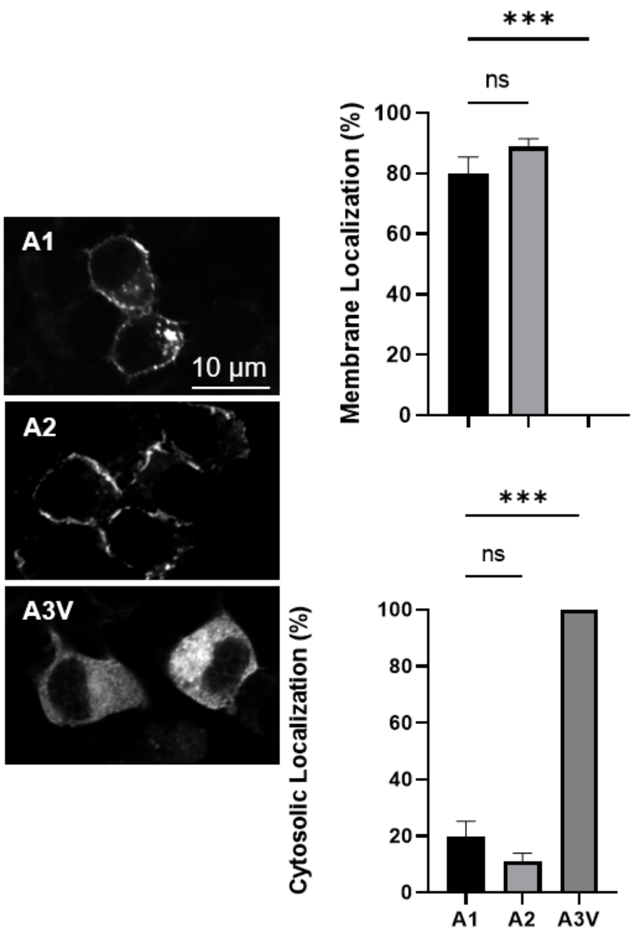

Supplemental Figure S1. A3V is expressed as a cytosolic protein, while A1 and A2, localize on the plasma membrane of N2A cells. N2A cells were transfected overnight with pEGFP-LC/A1 (A1), pEGFP-LC/A2 (A2), or pEGFP-LC/A3V (A3V), fixed with 4% paraformaldehyde, and imaged for EGFP fluorescence (ex^{488}nm/em^{509}nm). (**Left**) Representative images show the steady-state localization of A1, A2, and A3V. (**Right**) Percentage of EGFP membrane or cytosolically localized. Ten random fields were selected and counted for membrane (**Upper**) or cytosolic (**Lower**) localization. Mean and SEM was evaluated, with Ordinary one-way ANOVA with Dunnett's multiple comparisons test using A1 as the control column: NS= not significant, **=P<0.05, ***=P<.001.

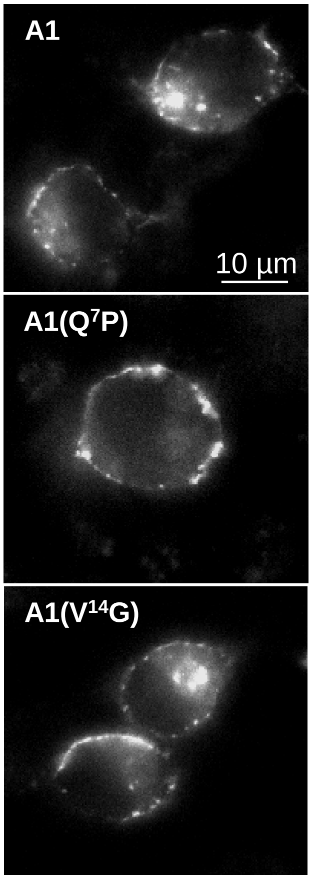

Supplemental Figure S2. Intracellular localization of A1 N Point mutations. After overnight transfections, N2A cells were fixed with 4% paraformaldehyde and imaged for EGFP fluorescence (excitation 488 nm/emission 509 nm). Representative images show the steady-state localization of EGFP-LC/A1 (A1), and EGFP-LC/A1(Q^7P) (A1(Q^7P)), and EGFP-LC/A1($V^{14}G$) (A1($V^{14}G$)).

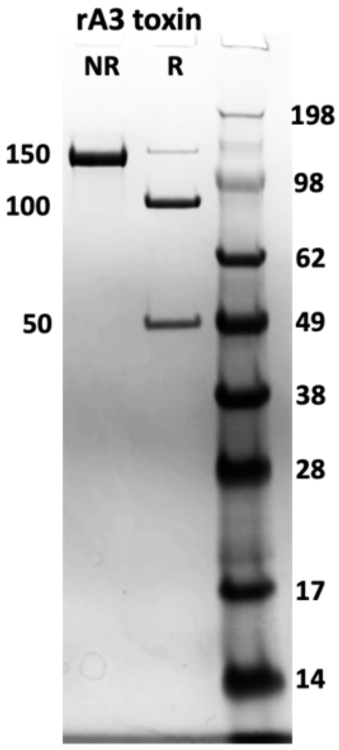

Supplemental Figure S3. SDS-PAGE analysis of BoNT/A3V. rBoNT/A3V (rA3V) was purified from *C. botulinum* strain Hall A *hyper* tox- and subjected to SDS-PAGE. Lanes: (**NR**), nonreduced full length rBoNT/A3V; (**R**), reduced rBoNT/A3V; (far right), showed migration of molecular weight marker Seeblue2.

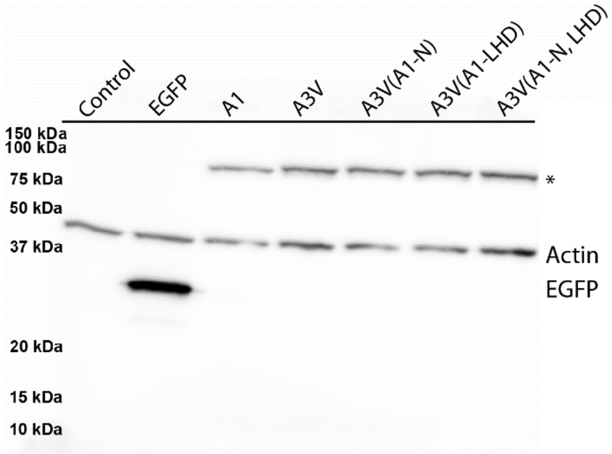

Supplemental Figure S4. Western blotting of GFP-A3V-A1. Cell lysates for N2A cells transfected with the DNA encoding the indicated EGFP-fusion protein (*) was subjected to 13.5% SDS-PAGE, transferred to a PVDF membrane, and probed for GFP with rat-anti-EGFP antibody 1:5000 followed by Goat-anti-rat IgG-HRP (1:20000) with Super Signal for detection as described in the methods section. Each GFP signal was detected in lysates expressing each EGFP-fusion protein, while lysates expressing EGFP alone yielded a (EGFP) reactive band at ~26 kDa. Actin (Actin) was probed as a loading control.
